# From Exosome Biogenesis to Absorption: Key Takeaways for Cancer Research

**DOI:** 10.3390/cancers15071992

**Published:** 2023-03-27

**Authors:** Nicolas Cheuk Hang Lau, Judy Wai Ping Yam

**Affiliations:** 1 Department of Pathology, School of Clinical Medicine, Li Ka Shing Faculty of Medicine, The University of Hong Kong, Hong Kong; 2 State Key Laboratory of Liver Research, The University of Hong Kong, Hong Kong

**Keywords:** exosome, extracellular vesicle, intercellular communication, biogenesis, targeting, regulation, cancer, endocytosis

## Abstract

**Simple Summary:**

Exosomes are nanometer-sized vesicles released by different cells that are important in the normal functioning of the body. In cancer, exosomes have been found to promote tumor growth and metastasis by carrying functional biomolecules and acting on different target sites in the body. Understanding the mechanism by which cancers modulate exosome secretion is crucial to studying cancer biology and developing new therapeutic approaches. Herein, we summarize the biological processes involved in the generation of exosomes, and the factors affecting the targeting and internalization of exosomes. By outlining the mechanisms regulating exosome dynamics, we link exosome secretion with characteristics of cancer cells and suggest methods for improving future research.

**Abstract:**

Exosomes are mediators of intercellular communication in normal physiology and diseases. While many studies have emerged on the function of exosomal cargoes, questions remain regarding the origin of these exosomes. The packaging and secretion of exosomes in different contexts modify exosomal composition, which may in turn impact delivery, uptake and cargo function in recipient cells. A mechanistic understanding of exosome biology is therefore crucial to investigating exosomal function in complex biological systems and to the development of novel therapeutic approaches. Here, we outline the steps in exosome biogenesis, including endosome formation, MVB formation, cargo sorting and extracellular release, as well as exosome absorption, including targeting, interaction with recipient cells and the fate of internalized exosomes. In addition to providing a framework of exosome dynamics, we summarize current evidence on major pathways and regulatory mechanisms. We also highlight the various mechanisms observed in cancer and point out directions to improve study design in exosome biology. Further research is needed to illuminate the relationship between exosome biogenesis and function, which will aid the development of translational applications.

## 1. Introduction

Extracellular vesicles (EVs) are a heterogeneous group of lipid bilayer-bound vesicles secreted by various cell types [1]. In recent years, many studies have emerged on the function of EVs in cancer and categorized them based on their morphology, cargo and donor cells [2,3]. Exosomes are a subset of EVs with a diameter of 30–150 nm and are known to regulate intercellular communication in normal physiology and diseases [4]. They carry cargoes, such as proteins, lipids, DNA and RNAs, including messenger RNA (mRNA) and noncoding RNA (ncRNA) [2], and mediate the exchange of functional contents between cells [5]. Studies have found that exosomes mediate various physiologic processes, such as neuronal communication, immune response and wound healing [6,7,8], while in cancer, exosomes are implicated in metastasis, immune evasion, therapy resistance and angiogenesis [3,9,10,11]. Notably, cancer cells utilize various mechanisms to regulate the amount and composition of exosomes to promote cancer development [12]. However, the unaccounted heterogeneity of secreted exosomes and the difficulty of harvesting EVs reproducibly reveal a bottleneck in our understanding of EV dynamics, both in vitro and especially in a biological system.

In ascending order of size, EVs are classified into exomeres, exosomes, ectosomes, apoptotic bodies and large oncosomes, of which exomeres and exosomes are classified into small EVs (sEVs), and the others are known as large EVs (lEVs) [13]. However, the observation that exomeres, which co-isolate with EVs, are non-membrane-bound particles has given rise to the term extracellular particles (EPs), used to classify the heterogeneous group of small, non-membrane-bound particles that includes exomeres [14]. While ectosomes, apoptotic bodies and large oncosomes are formed from the plasma membrane, exosomes are uniquely of endosomal origin and subject to numerous mechanisms of regulation and cargo loading [13].

In this review, we present the current knowledge on exosome biogenesis and relevant regulatory pathways. In addition, we provide a framework to understand exosome dynamics by highlighting mechanisms of exosome absorption. Finally, we suggest future directions of studying EV biogenesis to address research gaps and common experimental pitfalls. 

## 2. Mechanisms of Exosome Biogenesis

The pathway of exosome biogenesis is the most well studied among the subsets of EVs. Given their functional importance and the translational applications focused on exosomes exclusively, a mechanistic understanding of exosome biogenesis and absorption is necessary to forming functional hypotheses. 

**Figure 1:**
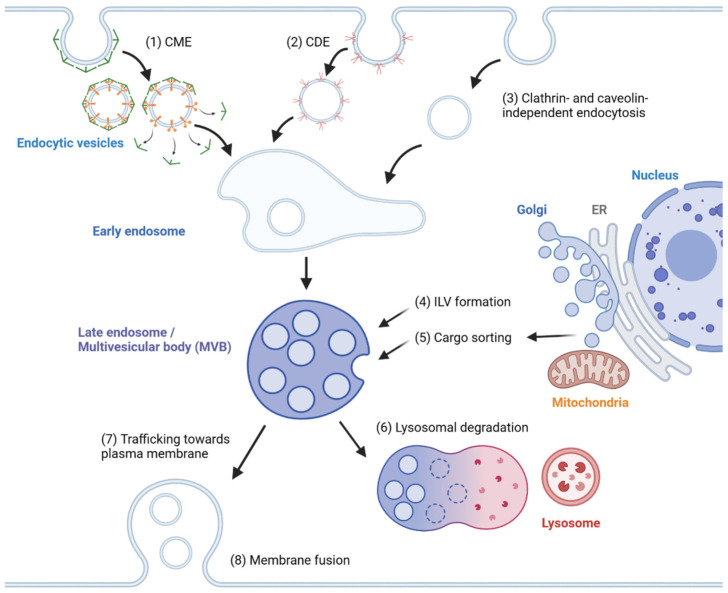
Overview of exosome biogenesis. Endocytic vesicles are generated by (1) clathrin-mediated endocytosis (CME), (2) caveolin-dependent endocytosis (CDE) and (3) clathrin- and caveolin-independent endocytosis. After the fusion of endocytic vesicles to form early endosomes, (4) ILVs are generated by ESCRT-dependent and ESCRT-independent pathways, resulting in the formation of multivesicular bodies (MVBs). (5) Cargoes are sorted into ILVs from various organelles, including the trans-Golgi network (TGN), endoplasmic reticulum (ER) and mitochondria. (6) MVBs can then fuse with lysosomes and undergo lysosomal degradation. (7) Alternatively, MVBs are trafficked towards the plasma membrane and (8) undergo membrane fusion for the extracellular release of ILVs as exosomes.

In general, exosome biogenesis is preceded by endocytosis, resulting in the formation of early endosomes. Several mechanisms cause the early endosome to incorporate cargo into intraluminal vesicles (ILVs), forming multivesicular bodies (MVBs) or late endosomes [15]. During maturation, MVBs may import cargoes from other organelles, including the trans-Golgi network (TGN), endoplasmic reticulum (ER) and other cytosolic compartments [16,17], further modifying the composition of ILVs. MVBs may fuse with lysosomes, resulting in degradation [2]. Alternatively, MVBs are transported to the plasma membrane, followed by membrane fusion, releasing ILVs as exosomes. The exosomes travel across extracellular space and reach a recipient cell, where several factors mediate the interaction between the exosome and the recipient cell [18]. Internalized exosomes are incorporated into early endosomes following the typical endosomal pathway and may be released again through recycling endosomes [19]. The overview of exosome biogenesis is depicted in Figure 1.

To summarize, exosome biogenesis consists of endosome formation, MVB formation, cargo sorting and extracellular release, while exosome absorption consists of targeting to the recipient cell, internalization and re-release. Each step is regulated by molecular pathways and environmental factors, leading to changes in the amount, composition, and eventually the dynamics of exosomes in a biological system. Despite sharing these common steps, exosomes isolated from biofluids are still highly heterogeneous due to cell-type-specific regulation of biogenesis and absorption, as well as the intracellular heterogeneity of MVBs and ILVs, the mechanisms for which will be highlighted in this section.

### 2.1. Endosome Formation

Early endosomes are the precursors of MVBs. Although a crucial condition for subsequent steps, it has yet to be sufficiently characterized in the context of exosome biogenesis. There are multiple pathways of endocytosis, including clathrin-mediated endocytosis (CME), caveolin-dependent endocytosis (CDE) and clathrin- and caveolin-independent endocytosis [20,21]. The mechanism for CME has been reviewed in detail elsewhere [20]. In short, a FCH domain only (FCHO) initiation complex is formed on the plasma membrane, forming a clathrin-coated pit. These proteins, along with adaptor protein 2 (AP2) and cargo-specific adaptor proteins, mediate cargo selection, followed by clathrin coat assembly and dynamin-mediated vesicle scission. The clathrin coat is finally disassembled by the ATPase heat shock cognate 70 (HSC70) and auxilin [20]. Other pathways include CDE, the clathrin-independent carriers/GPI-AP-enriched early endosomal compartments (CLIC/GEEC) pathway and the ARF6-associated pathway. Though the detailed mechanism of these pathways are not well defined, the reorganization of the actin cytoskeleton appears to play a central role in endosome formation, especially in pathways independent of clathrin or caveolin coating [21]. 

### 2.2. MVB Formation and Cargo Encapsulation

The endosomal sorting complex required for transport (ESCRT)-dependent pathway has been well studied for its involvement in ILV generation. The ESCRT machinery comprises ESCRT-0, -I, -II and -III complexes and Vps4 [22]. ESCRT-0 binds to ubiquitinated cargoes via the hepatocyte growth factor-regulated tyrosine kinase substrate (Hrs) subunit, while binding to phosphatidylinositol-3-phosphate (PI3P) on endosomal membranes, resulting in cargo enrichment on the limiting membrane of endosomes [22,23]. ESCRT-I is recruited to ESCRT-0 via the interaction of the ESCRT-I subunit, tumor susceptibility gene 101 (Tsg101) protein, with Hrs. This is followed by ESCRT-II binding to ESCRT-I. Together, they promote the inward budding of the endosomal membrane. Recruited by ESCRT-II, the ESCRT-III subunits Vps20, Snf7, Vps2 and Vps24 are sequentially assembled and promote the growth of the Snf7 polymer around the neck of the inward bud, driving further deformation, membrane fission and the formation of ILV [24]. The ESCRT-III subunit Did2 then replaces Vps24 and activates ATP hydrolysis by Vps4, resulting in the cleavage and disassembly of the ESCRT-III complex [24].

Numerous ESCRT-associated machinery cooperate with ESCRT in the formation of ILVs. Non-canonical ESCRT-dependent pathways, such as the Syndecan-Syntenin-Alix pathway and the his domain protein tyrosine phosphatase (HD-PTP) pathway also recruit ESCRT-III and VPS4 for the budding and scission of ILVs. The Syndecan–Syntenin–Alix pathway is initiated by the presence of the ubiquitous transmembrane protein Syndecan, recruiting Syntenin, which in turn binds Alix [25]. Proteins that are recognized by Syndecan, Syntenin or Alix are preferentially sorted into exosomes, such as fibroblast growth factor receptor (FGFR), lysyl-tRNA synthetase and tetraspanins, including CD9, CD63 and CD81 [25,26,27]. The phosphorylation of Syntenin by proteins and the cleavage by heparinase regulates the activity of the Alix-dependent pathway [28,29,30]. The HD-PTP pathway is initiated by the binding of HD-PTP to ESCRT-0, recruiting ESCRT-III and VPS4, thus re-entering the typical ESCRT-dependent pathway [31]. Components of the ESCRT pathway may interact with cargoes to promote their exosomal sorting; namely, the interaction of BAG6 and beta-catenin with Tsg101 and ESCRT-III, respectively, mediates their exosomal secretion [32,33].

The ESCRT-independent pathway involves a combination of lipid raft components, which together regulate the budding and formation of ILVs [34,35]. The neutral sphingomyelinase 2 (nSMase2)-ceramide pathway involves the conversion of sphingomyelin to ceramide by nSMase2, which allows ceramide to self-associate into raft-like macrodomains, leading to a negative membrane curvature that favors inward budding [36]. Microtubule-associated protein 1 light chain 3 (LC3), a well-known marker of autophagy, is located on the limiting membrane of MVBs and recruits the FAN protein, which promotes nSMase2-mediated ceramide production [37,38]. 

Other lipid raft components, such as cholesterol, caveolin-1 and flotillins, have been linked to exosome secretion and the ILV sorting of cargoes [12,39,40,41]. Proteins that promote cholesterol transfer to MVBs, such as ORP1L and STARD3, positively regulate the formation of ILVs [39,42]. While the precise mechanism of how cholesterol mediates ILV formation is unknown, cholesterol is found to promote flotillin 2 secretion in oligodendroglial cells, and flotillin-1 knock-down inhibits caveolin-1 secretion in PC-3 cells [41,43]. Thus, it is attractive to hypothesize whether cholesterol, caveolin-1 and flotillins constitute an alternative pathway of ILV formation, and whether they are related to ESCRT or ESCRT-independent machinery. However, cholesterol can have different effects on exosome release depending on the cell type; therefore, mechanistic studies using consistent cell types are preferred before a pathway can be confidently established.

Tetraspanins are integral membrane proteins that contribute to protein scaffolding in tetraspanin-enriched microdomains, which mediate adhesion and signal transduction [44]. CD63 has been found to interact with Apolipoprotein E to promote ILV formation in melanocytes independently of ESCRT and ceramide [45]. CD63 has also been used to promote reporter cargo loading in engineered exosomes [46]. Tetraspanin-6 (TSPN6) promotes exosomal secretion in HEK293 cells by interacting with Syntenin, likely co-opting the Syntenin–Syndecan–Alix pathway [30,47], though conflicting evidence suggests that TSPN6 inhibits exosome release in MCF-7 cells due to directing cargoes towards lysosomal degradation [48]. As common exosome biomarkers, CD9 and CD81 are enriched on exosomes, but their mechanisms are not well-defined [49]. While CD9 interacts with and mediates the exosomal secretion of CD10 [50], specific CD81 ligands appear to be abundant on tetraspanin-enriched microdomains [51], suggesting CD81 involvement in cargo sorting. Generally, tetraspanins mediate cargo loading into ILVs via molecular interaction, but their role in the formation of ILV, or its relevance or necessity for the ESCRT and ESCRT-independent pathways, has yet to be clearly investigated.

### 2.3. Cargo Sorting to MVBs

**Figure 2:**
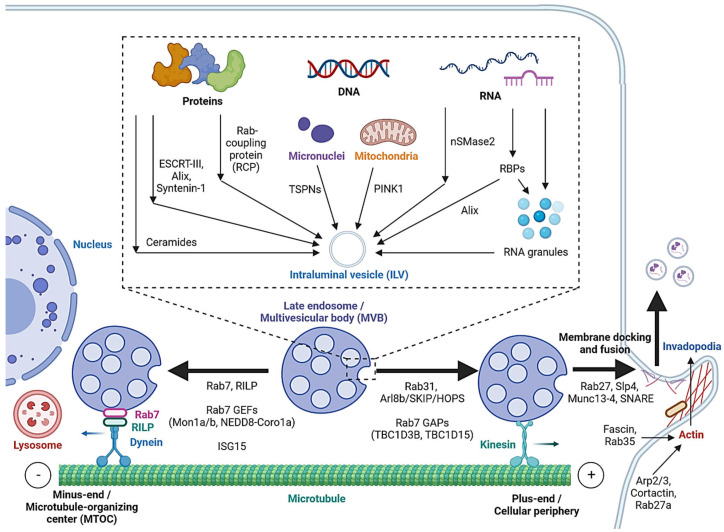
Cargo sorting mechanisms and the regulation of MVB trafficking and fusion with the cell membrane. Proteins are sorted into ILVs by associating with ESCRT-dependent and ESCRT-independent machinery and Rab coupling protein (RCP). Tetraspanins and PINK1 are involved in the loading of genomic DNA (gDNA) and mitochondrial DNA (mtDNA), respectively. RNA loading is regulated by nSMase2 and RNA-binding proteins (RBPs). Alix binds to the RBP Ago2 and promotes miRNA loading. RNA granules formed by liquid–liquid phase separation (LLPS) contain RNAs and RBPs and promote miRNA, lncRNA and circRNA loading. MVBs can be fated towards lysosomal degradation by translocation towards the microtubule minus-end or the perinuclear region. Alternatively, they are fated towards secretion by translocation towards the plus-end or the cellular periphery. Actin polymerization at invadopodia stabilizes MVB docking sites. MVB fusion is mediated by SNARE proteins, which are regulated by Rab27 and its effectors, Slp4 and Munc13-4.

Cargo sorting modulates the composition of ILVs and eventually exosomes. Exosomal cargo include proteins, amino acids, lipids, metabolites, DNA and RNAs, including messenger RNA (mRNA), microRNA (miRNA), long non-coding RNA (lncRNA), circular RNA (circRNA) and PIWI-interacting RNA (piRNA) [2,52]. The state of the donor cell can impact the proteomic and lipidomic profiles of the exosomal cargo [53]. As mentioned, protein cargoes are sorted into ILVs by interacting with components of the ESCRT-dependent or ESCRT-independent pathways, such as ESCRT-III, Alix, Syntenin-1 and ceramides [25,26,27,32,33,53]. In addition, Hsp90 alpha has been found to sort into ILVs by interacting with Rab coupling protein (RCP) [54] (Figure 2). 

While cell-free DNA (cfDNA) originates from various sources, a significant portion is localized on the surface or in the lumen of EVs [55]. Tetraspanins directly interact with cancer cell micronuclei, resulting in genomic DNA (gDNA) loading to exosomes [56]. Exosomal release also serves as a mechanism for removing harmful gDNA from the cytoplasm of healthy cells [57], whereas mitochondrial DNA (mtDNA) loading is initiated by mitochondrial damage, activating PINK1, which promotes the interaction of mitochondria and MVBs in an autophagy-independent manner [58].

Various mechanisms have been studied for RNA loading. nSMase2, which is involved in the ESCRT-independent pathway, has been reported to regulate the exosomal loading of miRNA in cancer cells [59,60]. Substance P (SP)/Neurokinin-1 Receptor (NK1R) signaling also increases miRNA expression and exosomal loading in colonocytes [61]. In addition, a number of RNA-binding proteins (RBPs), such as heterogeneous nuclear ribonucleoproteins (hnRNPs), Argonaute 2 (AGO2), Y-Box-Binding Protein-1 (YBX-1), Serine and Arginine-Rich Splicing Factor (SRSF1) and Major Vault Protein (MVP), bind to specific miRNAs motifs to facilitate their sorting to exosomes [5,62,63]. Meanwhile, hnRNPs also mediate the sorting of lncRNAs [64,65], and ESCRT-II and hnRNPA2B1 have been found to mediate the sorting of circRNAs [66,67]. Alix, well-known for its role in the ESCRT-dependent pathway, was also demonstrated to enrich miRNA in EVs via binding to Ago2 and miRNA [68].

In cells, biomolecular condensates, such as RNA granules, are formed by liquid–liquid phase separation (LLPS) [69]. Mechanistically, RNAs and RBPs contribute to RNA granule formation, which in turn contains components such as hnRNPA2 that facilitate miRNA, lncRNA and circRNA sorting into exosomes [69,70]. YBX-1 also forms LLPS-mediated condensates to promote miRNA loading into exosomes [71]. In addition, post-transcriptional modifications of miRNA sequences, in particular 3′ end adenylation and 3′ end uridylation, favor the cellular retention and exosomal release of miR-2909, respectively [72]. 

### 2.4. Extracellular Release

MVB translocation and fusion with the plasma membrane are required for the release of ILVs as exosomes. It primarily involves SNARE proteins that are well-known for their role in mediating membrane fusion events [73]. Various upstream mechanisms regulate the structure of SNAREs in different cell types that favor the fusion of MVBs with the plasma membrane, namely, the Fas/Fap-1/caveolin-1 cascade regulates the formation of SNARE containing SNAP25 and VAMP5 in mesenchymal stem cells [74], while the lncRNA HOTAIR regulates the formation of SNARE containing SNAP23 and VAMP3 in hepatocellular carcinoma [75]. 

As mentioned, MVBs can be fated towards the degradative pathway or the secretory pathway, based on their eventual fusion with lysosomes or with the cell membrane. Cytoskeleton filaments are crucial for MVB transport and docking. Actin reorganization, invadopodia formation and cortactin promote exosome secretion [76,77]. Cortactin binds to Arp2/3 and to actin filaments, leading to the stabilization of MVB docking sites and promote exosome secretion [77]. Coordinately, Rab27a promotes MVB docking by preventing Coronin1b localization to invadopodia, antagonization of cortactin and actin disassembly [77]. Rab35 binds and localizes the actin-bundling protein Fascin to actin, which facilitates actin cross-linking [78]. Accordingly, impeding Cortactin and Fascin-1 reduced EV secretion by regulating invadopodia formation [79]. Microtubules, on the other hand, play a role in the MVB fate by enabling movement towards their minus-ends or the plus-ends [80]. Trafficking towards the plus-end or the cellular periphery promotes membrane docking and the secretory fate of MVBs, whereas the minus-end is anchored in the perinuclear microtubule-organizing center (MTOC) and promotes the lysosomal degradative fate of MVBs.

Rab7 regulates MVB movement along cytoskeleton microtubules. Cargo movement towards the plus-end and minus-end of microtubules is mediated by kinesin and the dynein–dynactin complex, respectively [81]. Rab7 binds to its trafficking adaptor protein, Rab-interacting lysosomal protein (RILP), to recruit the dynein motor complex, thus promoting the transport of Rab7-positive vesicles towards the perinuclear region [82]. Cleavage of RILP due to inflammasome activation inhibits Rab7 binding to dynein motor complex, leading to kinesin-mediated movement of Rab7-positive vesicles to the cell periphery [83]. Thus, Rab7 appears to direct MVBs towards degradation, and its regulation alters the fate of MVBs. Rab31 recruits the GTPase-activating protein (GAP) TBC1D2B, which inhibits Rab7, resulting in increased MVB membrane fusion and exosome secretion [84]. Arl8b, also present on endosomal membranes, initiates the Arl8b/SKIP/HOPS cascade to recruit another GAP TBC1D15, which inhibits Rab7 [80]. As opposed to GAPs, the guanine nucleotide exchange factors (GEFs) Mon1a/b and NEDD8-Coro1a activate Rab7 by converting it to its GTP-bound state, resulting in lysosomal targeting and reduced EV secretion [85,86]. Additionally, induction of ISGylation via interferon-stimulating gene 15 (ISG15) overexpression was found to induce MVB protein degradation and colocalization with lysosomes [87].

Rab GTPases also control the MVB distribution, membrane docking and SNARE-mediated membrane fusion. Slp4 and Munc13-4 are downstream effectors of Rab27 that bind to and promote the formation of SNARE proteins to mediate the fusion of MVBs with the plasma membrane [88,89,90]. GEFs that regulate Rab27a/b, such as the DENN domain-containing protein Rab-3GEP (MADD) and FAM45A, promote endosomal maturation [91,92], and the stabilization of Rab27a by KIBRA also contributes to exosome secretion [93]. Rab27b regulates the trafficking of MVBs towards the plasma membrane by its effector Slac2b [88]. Rab11 and Rab35 enhance exosomal secretion in leukemia and oligodendrocytes, respectively [94,95]. Calcium ions (Ca^2+^) is required for the Munc13-4- and Rab11-mediated pathways of exosome secretion [96,97]. 

In summary, cytoskeleton components, such as actin filaments and microtubules, are crucial in MVB trafficking, docking and release. These processes are regulated by Rabs, which are in turn controlled by their GEFs and GAPs. Further study of Rabs and their effectors may help uncover viable mechanisms that modulate the volume and composition of secreted exosomes.

## 3. Mechanisms of Exosome Absorption

### 3.1. Exosome Transport and Targeting to Recipient Cells

**Figure 3:**
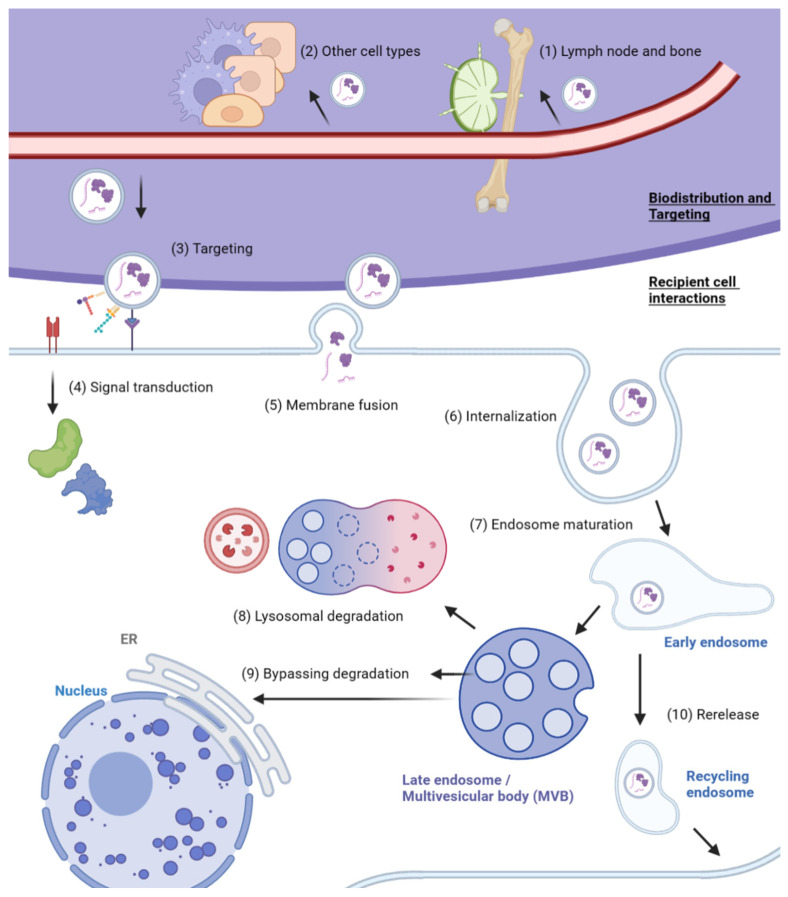
Overview of exosome biodistribution and uptake. The biodistribution of secreted exosomes is affected by (1) retention in lymph nodes and bone tissue and (2) competition from other cell types that vary in the rate of exosome uptake. Exosomes can be (3) targeted to recipient cells by receptor–ligand interaction, the presence of complex lipids on the exosome membrane, and recognition moieties from the donor cell type. Exosomes exert their functions in three ways—(4) activating signal transduction by receptor–ligand binding, (5) membrane fusion to release contents and (6) internalization via endocytic pathways. (7) Endosomes containing internalized exosomes eventually mature into multivesicular bodies (MVBs) and (8) undergo lysosomal degradation together with the exosomes. (9) Exosomal cargo may bypass degradation by passive diffusion, activation by acidification, RNA transfer to endoplasmic reticulum (ER) or release into the cytosol by exosome fusion with the endosomal membrane. (10) Internalized exosomes can also be re-released from early endosomes into the extracellular space via recycling endosomes.

After exosomes are released extracellularly, they are distributed systematically to facilitate local and distant intercellular communication. Exosomes have been found in numerous biofluids, including blood, serum, saliva, synovial fluid and breast milk [98]. The transport of exosomes is highly relevant to estimating their tissue-specific effector functions, and the progress on studying the biodistribution of exogenously administered exosomes has been reviewed in detail elsewhere [99]. In general, smaller EVs are less prone to retention in lymph nodes and bone tissue [14], and the intravenous injection of unmodified tumor-derived exosomes resulted in poor tumor accumulation of exosomes compared to intratumoral injection [100]. The route of administration, in addition to molecular targeting and donor cell type, are important considerations to modulate exosome biodistribution [101]. The overview of exosome uptake is depicted in Figure 3.

While all cells undergo non-selective exosome uptake, the difference in the exosome uptake capability of different cell types appears to be the main factor accounting for the organ-specific accumulation of tumor-derived exosomes [102]. On the other hand, several mechanisms also affect the delivery of exosomes to target tissues, including the specific interaction between exosomal surface molecules and cell surface receptors [103,104,105], complex lipids on the exosome surface [106,107] and recognition moieties favoring the same donor and recipient cell type [3,108,109]. This derives from the observation that cancer cell-derived exosomes preferentially fuse with their cell of origin compared to other cell types [110], and enhance the delivery of a chemotherapeutic agent to the donor cell type [111]. Specifically, the pattern of integrins and tetraspanins determines exosome targeting. ITGα_v_β_5_ mediates liver tropism, and ITGα_6_β_4_ and ITGα_6_β_1_ mediate lung tropism, while ITGα_4_β_7_ mediates exosome distribution to the small intestine [112,113]. Notably, blockage of ITGα_v_β_5_ and ITGα_6_β_4_ binding to fibronectin and laminin receptors, respectively, reduced their associated organ-specific exosome uptake and organotropic metastases [112]. As for tetraspanins, exosomes displaying the TSPAN8-alpha4 integrin complex are preferentially taken up by pancreatic cells [105], and CD63+ exosomes released by neuroblastoma specifically target neuronal dendrites, as opposed to CD63- exosomes, which target neurons and also glial cells [114]. Other exosomal proteins, such as integrin-associated protein (CD47), CD55 and CD59 facilitate immune evasion, leading to extended time in the circulation [115,116].

### 3.2. Interaction with Recipient Cells and Internalization

**Figure 4:**
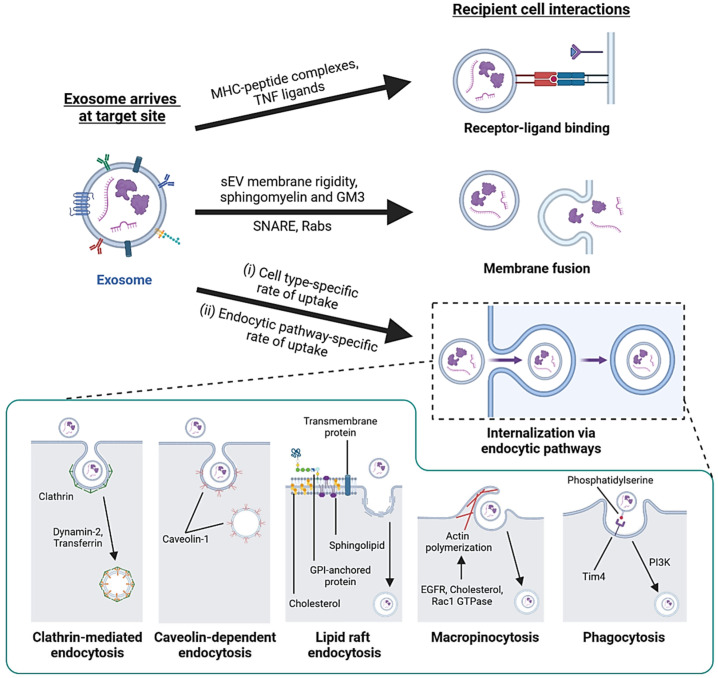
Interactions of exosomes with recipient cells. Exosomes carrying MHC–peptide complexes or TNF ligands undergo receptor–ligand interaction with recipient cells to activate immune cells or trigger apoptosis in cancer cells. Exosomes that have high membrane rigidity and enrichment in sphingomyelin and GM3 have increased fusion efficiency. SNARE and Rab proteins are required for membrane fusion. Most exosomes are taken up by internalization. Different cell types differ in overall exosome uptake efficiency, and each cell may use more than one endocytic pathway for exosome uptake in parallel. Endocytic pathways, such as clathrin-mediated endocytosis (CME), caveolin-dependent endocytosis (CDE), lipid raft endocytosis, macropinocytosis and phagocytosis, contribute to exosome uptake.

After transportation to recipient cells, exosomes can function in three ways—internalization, membrane fusion or receptor–ligand binding (Figure 4). Internalization is a temperature-sensitive process where cells rapidly uptake exosomes via endocytic pathways that subject exosomes to various internalized fates [117]. To a lesser extent, exosomes fuse with the plasma membrane and directly release their contents into the cytosol. Additionally, ligands on the exosome surface can bind with cell surface receptors to trigger signaling cascades in target cells [118]. Exosomes interact with cell surface receptors by carrying molecules, such as MHC–peptide complexes and tumor necrosis factor (TNF) ligands, which usually function as signal transduction in the immune response and induction of apoptosis in cancer cells [118,119]. Exosome fusion with the plasma membrane involves the formation of a hemi-fusion stalk between the lipid bilayers, followed by stalk expansion and opening of the fusion pore [120,121]. SNAREs and Rab proteins are involved in membrane fusion [122]. Moreover, membrane fusion is promoted for exosomes secreted by tumor cells in acidic pH conditions due to the increased exosomal membrane rigidity and the high content of sphingomyelin and ganglioside GM3 lipids that mediate membrane fusion [107]. 

Most exosomes are taken up through internalization [121,123], utilizing endocytic pathways, such as CME, lipid raft endocytosis, macropinocytosis and other mechanisms. As mentioned in Section 2.1, CME involves the formation of clathrin-coated pit with AP2 adaptor proteins, which deforms and favors inward membrane budding and dynamin-mediated scission, followed by uncoating to form endocytic vesicles that fuse with and deposit contents into endosomes [20,124]. Inhibition of AP2 or dynamin-2 function has been reported to reduce exosome uptake [117,125]. Lipid raft endocytosis is a possible route of exosome internalization. These rafts are enriched in sphingomyelin, cholesterol and flotillins, which facilitate clathrin- and caveolin-independent endocytosis [126,127]. Inhibiting the biosynthesis of sphingolipids or the intracellular transport of cholesterol results in reduced exosome uptake [128,129]. Macropinocytosis involves the formation of membrane invaginations by lamellipodia, which then pinch off to form macropinosomes [121]. Exosome uptake by macropinocytosis is not sensitive to exosomal composition due to the lack of direct contact between the cell and internalized material [130]. The mechanism is regulated by Rac1 GTPase, Actin and cholesterol [130]. Growth factors also promote micropinocytosis, such as that induced by EGFR signaling in cancer cells expressing Ras [131,132].

Exosome uptake by CDE is possible, but conflicting evidence exists. Reduction in caveolin-1 by genetic knockdown leads to reduced exosome uptake [133], while genetic knockout increases exosome uptake in fibroblasts [134]. Exosome uptake by phagocytosis is primarily utilized by immune cells and explains the higher rate of exosome uptake in macrophages [125,135]. Cargoes internalized in phagosomes are thought to be directed towards lysosomal degradation [136]. Phosphatidylinositol-3-kinase (PI3K) is required for forming phagosomes, and its pharmacological inhibition reduces exosome uptake in phagocytic cells [125,137]. Phosphatidylserine (PS) enriched on exosomes is recognized by the TIM4 receptor on macrophages and facilitates phagocytosis [125]. Consequently, the masking of PS on exosomes by annexin-V treatment reduces the exosomal uptake by immune cells [7,138]. 

### 3.3. Fate of Internalized Exosomes

Following internalization, endosomes containing exosomes mature into MVBs, eventually undergoing lysosomal degradation in the target cell [139]. However, some cargoes bypass degradation by passive diffusion across the exosomal membrane [140], or if the exosomal cargo function is activated by acidification, as in the case of transforming growth factor (TGF) beta-1 [141]. In addition, the interaction of ER with endosomes allows the transfer of exosomal RNAs into the ER and facilitates the translation of exosomal RNA [142]. Cargoes may also be released into the cytosol by membrane fusion between exosomes and endosome [123], which can be enhanced by molecules, such as the exosomal surface lipid lysobisphosphatidic acid (LBPA) [143]. 

Recycling endosomes, which typically serve to return endocytosed plasma membranes to the cell surface, can facilitate the re-release of exosomes back to the extracellular space, allowing the penetration of exosomes across multiple cell layers [144,145]. The exosome biogenesis and absorption discussed are summarized in Table 1 and Table 2.

## 4. Relevance of Exosome Secretion and Uptake in Cancer and Cancer Therapy

### 4.1. Regulation of Exosome Secretion in Cancer

Exosomes promote cancer development by priming premetastatic niches, evading immune surveillance and developing resistance to therapy [3,9,10,11]. To this effect, various mechanisms are utilized by cancer cells to regulate the amount and composition of exosomes.

Exosomal biogenesis is directly affected by the aberrant expression of exosome biogenesis machinery. For the formation of MVBs, the proteins Hrs, Hsp90 and Syntenin act as oncogenes to promote exosome release and promote cell invasion in cancer cells [76,146,147]. Notably, Vps4A and HD-PTP may act as tumor suppressors, either by promoting the exosomal secretion or the lysosomal degradation of oncogenic proteins, resulting in reduced cell proliferation and metastasis in donor cells [31,33]. Dysregulation of molecular signaling pathways in cancer have also been found to modulate exosome release, such as the Ras/Raf/MEK/ERK [148], Ca^2+^ [97], glutamine [149], p53 [150], mTOR [151], STAT3 [152] and GPCR signaling pathways [153]. 

In addition, miRNAs can negatively regulate the expression of biogenesis machinery [154], while lncRNAs can regulate exosome release by regulating gene expression [75,155] or promoting the colocalization of SNARE components [75]. Post-transcriptional modifications of biogenesis machinery, such as phosphorylation [28], GlcNac modification [156], mono-ubiquitination [157] and SUMOylation [65], are also mechanisms of modulating exosome biogenesis in cancer cells. Extracellular environment and stresses can also affect exosome secretion. The tumor microenvironment (TME) is observed to regulate exosome secretion by hypoxia- and acidity-related regulation of cellular signaling [107,158]. Lactate released by cancer cells in the TME also recruits tumor-associated macrophages (TAMs), which secrete exosomes with tumorigenic cargo [159].

### 4.2. The Role of Exosome Targeting in Cancer and Drug Delivery

Cancer-specific alterations to exosome biogenesis are relevant with respect to how they modulate exosome function. Tumor-derived exosomes produced in the low-pH tumor microenvironment have higher membrane rigidity and increased sphingomyelin content [107]. They are more readily taken up by tumor cells via membrane fusion, which promotes the paracrine diffusion of malignancy [107]. Tumor cells also preferentially uptake exosomes from donor cells of the same type due to conservation of cellular signatures [108], contributing to the horizontal transfer of cargo between tumor cells.

In addition to the variety of tumorigenic and metabolic effects induced in tumor cells, exosomes also mediate the crosstalk of tumor cells, the extracellular matrix, stromal cells and immune cells [160]. Exosomes regulate the macrophage polarization between M1 and M2 phenotypes, and can induce the transformation towards M2 macrophages that promote tumor growth [161]. Tumor-derived exosomes also act on T cells, B cells and natural killer (NK) cells, activating immune responses while contributing to immune evasion [160]. 

The study of exosome targeting and uptake is especially relevant to their use as drug delivery vehicles. To generate exosomes that display desired surface molecules, genetic engineering or chemical engineering approaches have been employed. Genetic engineering refers to the incorporation of ligand-expressing plasmids into donor cells, while chemical engineering involves chemical conjugation or electroporation of collected exosomes [62,162]. Genetic engineering is more favorable towards exosome integrity, as it utilizes the natural biogenesis pathways, whereas direct modification methods may negatively impact the stability of the membrane structure, as well as the endogenous and exogenous targeting molecules, which hinders the targeting effectiveness of the produced exosomes.

Generally, genetic vectors encoding for a fusion product between an exosomal membrane component and a targeting moiety have been used to modify exosomes from donor cells. For example, lysosomal-associated membrane protein 2b (Lamp2b) fusion with αv integrin-specific iRGD peptide has been used to target breast cancer cells [163], and Lamp2b fusion with neuron-specific rabies viral glycoprotein (RVG) peptide targets acetylcholine receptors in the brain [103]. Platelet-derived growth factor receptor (PDGFR) on the exosomal surface has also been fused with antibodies targeting CD3 and EGFR to produce synthetic multivalent antibody-retargeted exosomes (SMART-Exos) which simultaneously target T cells and breast cancer cells [164]. In addition, glycosylphosphatidylinositol (GPI) anchor peptides, inherently enriched on the exosomal membrane, have been fused with anti-EGFR nanobodies to facilitate targeting to cancer cells [165,166].

### 4.3. Cancer Exosomes as a Therapeutic Target

Cancer-specific dysregulation of exosome biogenesis has given rise to the pharmacological inhibition of exosome machinery as a potential avenue of treatment. To inhibit exosome biogenesis and release, pathways, such as nSMase2 [167], Syntenin [168] and Ras signaling [148,169], have been targeted. GW4869 is a well-studied nSMase2 inhibitor that inhibits the ESCRT-independent nSMase2-ceramide pathway [167]. It sensitizes anti-programmed death-ligand 1 (PD-L1) therapy by inhibiting exosomal secretion of PD-L1 by cancers [170], and also reduces glioma progression by promoting the cellular retention of tumor-suppressing miRNA [171]. While GW4869 inhibits exosome release, it stimulates multivesicle release in breast adenocarcinoma cells [172], suggesting its nonspecific action may hinder the inhibition of overall EV release when considering its potential for clinical translation. Manumycin A is an inhibitor of Ras farnesyltransferase (FTase), thereby inhibiting Ras/Raf/ERK1/2 signaling required for the ESCRT-dependent pathway and reduced EV release in prostate cancer cell lines [148].

Besides exosome biogenesis, exosome transport can also be inhibited via various approaches. HER2-positive EVs induce the formation of premetastatic niches and promote resistance to breast cancer therapy [173]. A hemofiltration system utilizing customizable affinity matrices comprising exosome-binding antibodies, lectins and aptamers was proposed to remove HER2-positive cancer-derived EVs [173]. In humans, regular hemodialysis was confirmed to reduce circulating EVs via adsorption to the dialysis membrane, though this reduction is not specific to the EV origin [174]. Alternatively, antibodies can be used to interfere with exosome-mediated disease progression. Treatment with anti-CD9 and anti-CD63 antibodies increased the macrophage internalization of EVs in vitro and decreased the lung and lymph node metastasis of breast cancer in mice [175]. However, this approach is not specific to tumor-derived EVs and may interfere with normal exosome physiology. In the future, antibodies against tumor-specific exosomal markers may reduce off-target effects, and their viability should be explored. The combination of exosome-depleting adjuncts with chemotherapy agents may increase the efficacy, reduce metastasis and minimize chemoresistance.

Finally, EV uptake inhibitors have been explored to reduce cancer-derived EV uptake by both cancer and non-cancer cells, with the aim of reducing EV-mediated chemoresistance, proliferation and metastasis [176]. Dynasore is a non-competitive inhibitor of dynamin1, dynamin2 and dynamin-related protein 1 (Drp1), which are required for production of clathrin-coated endocytic vesicles during CME [177]. Dynasore is commonly used to inhibit CME [178] and reduces the uptake of exosomes in vitro [179]. However, dynasore has non-specific effects by regulating actin bundling [178], which may indicate its potential role in exosome release, though this is poorly characterized. Ikarugamycin (IKA) specifically inhibits CME and not other endocytic pathways, and it acts by targeting transferrin receptors involved in CDE [180]. Its inhibitory effect is potent, fast-acting and reversible, and thus, it is suitable for research use; however, its specificity and toxicity have not been studied sufficiently to assess its viability for clinical translation. Heparin sulfate proteoglycans (HSPGs), such as Syndecan, are internalizing coreceptors that modulate cancer-derived exosome uptake [181]. Heparin is a clinically used anticoagulant that competitively inhibits HSPGs on cells and was found to a cause dose-dependent and charge density-dependent reduction in exosome uptake [182]. Alternatively, healthy pancreatic tissue surrounding pancreatic cancer releases the lectin Reg3β, which binds to exosomal surface glycoproteins and reduces the uptake of cancer-derived exosomes in vivo [183]. Functionally, Reg3β reduces EV-mediated cancer cell migration and macrophage phenotypic switching in vitro [183], suggesting its potential role in reducing cancer-derived EV uptake by both cancer and non-cancer cells. 

To select drugs for clinical translation, the solubility and potency of inhibitors should be adequately tested [167]. Currently, there are difficulties in clinical translation in terms of drug efficacy, drug specificity and gaps in mechanistic knowledge. While some drugs target only one protein in EV biogenesis, other drugs may have multiple targets, such as Manumycin A, which targets both Ras farnesyltransferase and nSMase2. Dynasore may target both EV release and EV uptake via different mechanisms. While inhibitors that target multiple pathways may be more efficacious, their downstream effects are more difficult to control precisely. In addition, various pathways of EV release and EV uptake act in parallel, with each pathway contributing to a portion of EV flow. As such, a single EV inhibitor may not be sufficient to inhibit EV release or uptake completely. GW4869 and manumycin A used together produced significantly better EV reduction than manumycin A alone [148], suggesting that multiple pathway inhibitors are needed to completely block EV release. On the other hand, this may support the notion of designing specific therapies that inhibit disease-specific EV flow with significantly less alteration of the normal physiological roles of EVs.

However, the observation that promoting MVB formation can be either oncogenic (via the activation of Hrs and Syntenin) or tumor suppressing (via the activation of Vps4A and HD-PTP) in a molecule-specific and cell-type-specific manner is a major barrier to translational application. This confusion reflects an insufficient understanding of the mechanism of action of the therapeutic compounds or of the biological pathway itself. In addition, despite the identification of key machinery in exosome biogenesis, conflicting evidence remains regarding the upstream regulators of the mechanisms [37,38]. Therefore, the identification of novel effectors and further understanding of exosome mechanisms in pathway-specific and cell-type-specific contexts will aid the identification of new therapeutic approaches.

## 5. Challenges and Perspectives for Designing Studies on Exosome Biology

In the recent decade, the heterogeneity of exosomes has been brought to light. As a result, exosomes have been further divided into small exosomes (Exo-S, 30–90 nm) and large exosomes (Exo-L, 90–150 nm) [184]. Even within the same MVB, subpopulations of ILVs can be produced via different mechanisms. This gives rise to exosomal subpopulations with distinct biological and physical properties [185]. The basic differentiation of exosomes from other EVs relies on the isolation and characterization methods advised by ISEV [186], including nanoparticle tracking analysis (NTA), transmission electron microscopy (TEM) and western blotting. Eventually, techniques may be required to further differentiate between the physically and compositionally distinct exosomal subpopulations, such as flow cytometry and proteomic techniques [1]. These methods, however, suffer from the lack of a standardized technique and the lack of consistent and specific markers for EV subpopulations [1].

Proteins should be tested for their involvement with major biogenesis pathways in order to gain further understanding of the interaction or independence of exosome biogenesis mechanisms. For example, the use of specific pathway inhibitors has been used to block endocytic pathways for the study of exosome uptake in vitro [121].

Given the multiple downstream functions of exosomal machinery, gain-of-function or loss-of-function studies for studying exosome secretion may alter other cellular processes besides the pathway of interest [2,18], which may indirectly impact exosome secretion, potentially yielding false-positive results. Experimental reproducibility is also negatively impacted without proper standardization and documentation of culture conditions, exosome collection protocols and pre-analytical variables [1,187], the effects of which have yet to be quantified scientifically. 

Reliable methods are required for studying exosome uptake. One method is the loading of EVs with luciferin substrate, followed by the administration to luciferase-expressing cells and the measurement of bioluminescence, which studies the transfer of exosomal contents into cytosol [121,137]. To visualize the interaction between exosomes and cells, studies may also stain exosomes with fluorescent lipophilic membrane-binding dyes, which can be subsequently measured by fluorescence microscopy or flow cytometry [121]. However, these methods merely track exosomes and their surface lipids and cannot be used to visualize the fate of intracellular cargoes themselves. 

While exosome bioengineering provides a promising future for targeted drug delivery, several barriers stand in the way of its translation. Genetic modification of donor cells applies only to targeting molecules and fusion products that can be encoded within a genetic vector. The feasibility of modifying exosomes from patient-specific donor cells via a genetic approach is also uncharacterized [188]. Furthermore, targeting moieties intended for exosomal incorporation can be degraded within donor cells, though this can be overcome via the addition of glycosylation motifs to stabilize the targeting peptide [189]. On the other hand, chemical engineering overcomes several of these limitations, at the cost of requiring strict reaction conditions that ensure the stability of the exosome membranes, intrinsic targeting molecules and extrinsic targeting molecules [188]. 

## 6. Conclusions

Given the complex networks of protein interactions, individual proteins may exert more than one effect on the exosomes produced. As mentioned, some proteins have dual roles by simultaneously promoting and inhibiting different mechanisms in EV biogenesis. Moreover, the function of a protein in exosome biogenesis can be cell-type-specific. Homologs from the same protein family are responsible for loading different cargos into ILVs. Therefore, observations from a particular cell type, protein and conditions should be well-documented and cautiously interpreted when applied in other contexts. Currently, despite being aware of the heterogeneity of exosomes and MVBs, it is unknown which mechanisms can regulate such heterogeneity and the extent to which these mechanisms are cell-type-specific.

Recent studies in the regulation of exosome release have enabled the framework of exosome biogenesis and absorption to be identified; thus, we have attempted to unite current evidence and point out common elements in exosome biology in this review. However, cell-type-specific pathways and interactions between mechanisms remain ambiguous. Delineating these mechanisms will be crucial to the conceptualization and development of novel, specific and effective therapeutic approaches. However, exosome isolation methods and the analysis of the exosome composition are unstandardized. Establishing reliable methods in characterizing exosome subpopulations is needed for overcoming the current bottleneck. 

Numerous translational opportunities have been proposed for exosomes and their biogenesis. Exosomes have been suggested as biomarkers for the diagnosis and evaluation of cancer progression, but heterogeneity has been an obstacle for the analysis of EV composition and obstructing the detection of disease-specific exosomes. Additionally, exosomes can act as drug delivery vectors for cancer therapy. Exosome engineering approaches can incorporate targeting moieties that improve organ-specific targeting. However, the low yield and compositional heterogeneity is an obstacle to harvesting and controlling the side effects of engineered EVs. Further study in exosome biology and bioengineering is needed to optimize the specific targeting and efficient manufacturing of exosomes for translational medicine.

## Figures and Tables

**Table 1:** Steps in exosome biogenesis and associated pathways.

Steps	Pathways	Mechanisms	References
Exosome biogenesis			
Endosome formation	Clathrin-mediated endocytosis	FCHO, AP2, adaptor proteins, Dynamin, HSC70, auxilin	[20]
	Caveolin-dependent endocytosis	Caveolin-1	[21]
	Clathrin- and caveolin-independent endocytosis	CLIC/GEEC; ARF6; Actin	[20,21]
MVB formation	ESCRT-dependent pathway	ESCRT-0/I/II/III, VPS4	[22,23,24]
	Non-canonical ESCRT-dependent pathways	Syndecan-Syntenin-Alix, heparinase; HD-PTP	[25,28,29,30,31]
	ESCRT-independent pathway	LC3, nSMase2, ceramide; cholesterol, caveolin-1, flotillins; TSPNs (CD63, TSPN6, CD9, CD81)	[34,35,36,37,38] [39,40,41,42,43] [45,46,47,48,49,50,51]
Cargo sorting	Protein-sorting pathways	ESCRT or ESCRT-independent pathways; Rab coupling protein	[25,26,27,32,33,53,54]
	DNA-sorting pathways	TSPN interaction with micronuclei; PINK1	[56,58]
	RNA-sorting pathways	nSMase2, SP/NK1R, RBPs, ESCRT-II, Alix; LLPS, 3′ end adenylation or uridylation	[5,59,60,61,62,63,64,65,66,67,68] [69,70,71,72]
Extracellular release	SNARE-mediated membrane fusion	Fas/Fap-1/caveolin-1, lncRNA HOTAIR	[74,75]
	Actin reorganization	Cortactin, Arp2/3, Rab27a, Coronin1b; Rab25, Fascin	[76,77,78,79]
	Microtubule-mediated transport	Rab7, RILP; Rab31, Arl8b/SKIP/HOPS, Rab7 GAPs (TBC1D2B, TBC1D15), Rab7 GEFs (Mon1a/b, NEDD8-Coro1a); ISG15	[80,81,82] [83,84,85,86] [87]
	Rab-regulated trafficking and fusion	Rab27a/b, Rab27 GEFs (MADD, FAM45A), KIBRA, Slp4, Munc13-4; Rab11; Rab35, Ca^2+^	[88,89,90,91,92,93] [94,95,96,97]

Abbreviations: TSPN, tetraspanin; SP/NK1R, substance P/neurokinin-1 receptor; RBP, RNA-binding proteins; LLPS, liquid–liquid phase separation; lncRNA, long non-coding RNA; GAP, GTPase activating protein; GEF, guanine nucleotide exchange factor.

**Table 2:** Steps in exosome absorption and associated factors.

Steps	Factors/Processes	Mechanisms	References
Exosome absorption			
Exosome biodistribution and organ-specific accumulation	Retention in lymph nodes and bone	Exosome size	[14]
	Route of administration	Intratumoral, intravenous, etc.	[99,100,101]
	Cell type-specific rate of exosome uptake	Cell type-dependent factors	[102]
Targeting to recipient cell	Receptor–ligand interaction	RVG, SDF-1; integrins (α_v_β_5_, α_6_β_4_, α_6_β_1_, α_4_β_7_), TSPNs (TSPAN8, CD63), CD47	[103,104,105] [112,113,114,115,116]
	Lipid targeting	Phosphatidylethanolamine, sphingomyelin	[106,107]
	Cell-type-specific exosome uptake	Recognition moieties from the donor cell type	[108,109,110,111]
Recipient cell interactions	Signal transduction via receptors	MHC-peptide complex, TNF receptors	[118,119]
	Membrane fusion	Rabs, SNARE; membrane rigidity and lipid content	[107,122]
	Clathrin-mediated endocytosis	Dynamin-2; transferrin	[117,124,125]
	Lipid raft endocytosis	Cholesterol, sphingolipid, flotillin	[126,127,128,129]
	Macropinocytosis	Rac1 GTPase, actin, cholesterol, EGFR	[130,131,132]
	Caveolin-dependent endocytosis	Caveolin-1	[133,134]
	Phagocytosis	PI3K, phosphatidylserine	[125,135,136,137,138]
Internalized exosomes	Lysosomal degradation	Trafficking to lysosomes (e.g., Rab7)	[80,139]
	Bypassing lysosomal degradation	Passive diffusion; cargo activation by acidification; RNA transfer to ER; fusion with endosomal membrane (e.g., LBPA)	[108,140,141,142,143]
	Re-release	Sorting to recycling endosomes	[144,145]

Abbreviations: RVG, rabies viral glycoprotein; TSPN, tetraspanin; ER, endoplasmic reticulum; LBPA, lysobisphosphatidic acid.
